# Risk factors associated with return sepsis admission following emergency department discharge with infection

**DOI:** 10.1016/j.ajem.2025.07.059

**Published:** 2025-07-27

**Authors:** Alice Y. Chen, Matthew Allison, Michael Puskarich, Gary M. Vilke, Pam Taub, Michael H. Criqui, Gabriel Wardi, Victor Nizet, JoAnn Trejo, Edward M. Castillo, Jesse Brennan, Christopher Coyne

**Affiliations:** a Department of Emergency Medicine, School of Medicine, University of California San Diego, San Diego, CA, United States; b Division of Preventive Medicine in the Department of Family Medicine, School of Medicine, University of California San Diego, San Diego, CA, United States; c Department of Emergency Medicine, University of Minnesota, Minneapolis, MN, United States; d Department of Cardiovascular Medicine, School of Medicine, University of California San Diego, San Diego, CA, United States; e Department of Pediatric Medicine, School of Medicine, University of California San Diego, San Diego, CA, United States; f Department of Pharmacology, School of Medicine, University of California San Diego, San Diego, CA, United States

**Keywords:** Sepsis, Risk factors, Risk stratification, Prediction model, Outpatient, Return admission, Sepsis admission

## Abstract

**Introduction:**

Despite sepsis having growing awareness nationally, efforts to reduce the public health impact of sepsis have lagged. Although there are known pathophysiologic mechanisms and preventive strategies, sepsis is rarely approached as a predictable or preventable condition. Predicting who will develop sepsis in patients with infection still remains a challenge. This study examined modifiable and nonmodifiable risk factors associated with patients initially discharged home with an infection and had future sepsis-related admissions within 7 days of the index Emergency Department (ED) visit.

**Methods:**

We conducted a multi-center retrospective cohort analysis of adults presenting to two university hospital EDs. The inclusion criteria encompassed adult patients who were discharged from the ED at their index visit with discharge diagnosis (ICD 10-CM code) of pneumonia, urinary tract infection (UTI), and/or cellulitis and who returned for hospital admission within 7 days of the index visit due to sepsis, severe sepsis without septic shock, and/or septic shock. Using multivariate regression, risk factors that predict return sepsis admission within 7 days of ED index visit were evaluated, and a 7-day return sepsis admission model was constructed. The predictive power of the model was measured by c-statistic.

**Results:**

Among 10,179 unique ED patients, return sepsis admissions within 7 days occurred in 113 visits (1.11 % of discharged patients). Statistically significant risk factors among patients with infection associated with subsequent sepsis admission in the chosen model were Cardiovascular Disease (OR 2.07 95 % CI 1.26–3.42), Hypertension (OR 2.21 95 % CI 1.37–3.56), Chronic Kidney Disease (OR 1.80 95 % CI 1.11–2.91), Cancer (OR 2.22 95 % CI 1.43–3.45), Male (OR 1.67 95 % CI 1.13–2.45), arriving in an ambulance (vs. walk in OR 2.55 95 % CI 1.46–4.44), higher heart rate (OR 1.29 95 % CI 1.16–1.45), and higher temperature (OR 1.23 95 % CI 1.05–1.45), Hyperlipidemia was protective (OR 0.56 95 %CI 0.34–0.91). The c-statistic of our chosen model was 0.77 (95 % CI 0.73–0.81). The Hosmer-Lemeshow test for our logistic regression model resulted in a chi-square value of 7.23 with 8 degrees of freedom with a *p*-value of 0.51. This suggests that our model fits the data well.

**Conclusion:**

Our findings may be used to risk stratify and guide outpatient disposition decisions for ED patients with infection and to determine which patients need to be more closely monitored in the outpatient setting following ED discharge.

## 1. Introduction

Sepsis is the body’s overwhelming and life-threatening response to an infection [1]. The Centers for Disease Control and Prevention (CDC) declared sepsis as a medical emergency in 2016 [2], as recent estimates have suggested its incidence to be approximately 48.9 million incident cases annually worldwide [3]. Sepsis infection incurred more than $24 billion in total US hospital costs in 2017, making it one of the most expensive inpatient diagnoses in the United States and the leading cause of inpatient deaths [3–6].

Major public health improvements in the U.S. have stemmed from evidence-based strategies to detect, stratify, and reduce risks for common conditions like cardiovascular disease and stroke, such as the Heart Score developed to assess and stratify cardiovascular events in the Emergency Department and CHA2DS2-VASc score for stroke. We hope to inform clinicians about factors that influence sepsis readmissions after initial infection. Despite sepsis having growing awareness nationally, efforts to reduce the public health impact of sepsis have lagged. Although there are known pathophysiologic mechanisms and preventive strategies, sepsis is rarely approached as a predictable or preventable condition. Predicting who will develop sepsis in patients with infection still remains a challenge.

Prior studies have linked sepsis to demographic and lifestyle factors such as older age, male sex, lower education, and tobacco and alcohol use, alongside co-morbid conditions such as chronic lung disease, peripheral artery disease, chronic kidney disease, diabetes, deep vein thrombosis, coronary artery disease, and hypertension [7–9]. It remains unclear what characteristics of patients with infection presenting in the ED later develop sepsis and require hospitalization. Identifying risk factors for sepsis among patients presenting with infection in the ED could help prioritize those at high risk for developing sepsis hospital admission and requiring close outpatient monitoring after initial infection diagnosis. To address this, we compared the characteristics of patients discharged from the ED with infections (i.e., pneumonia, UTI, cellulitis) who returned within 7 days with a sepsis-related admission versus those who did not return for sepsis-related admission.

## 2. Methods

### 2.1. Study design and setting

We conducted a retrospective cohort study on adult patients who visited the UC San Diego Health ED across three calendar years. We enrolled patients who presented to the ED from January 1, 2019 through February 28, 2022, and evaluated them to see if they returned to the ED for sepsis-related admissions within seven days of initial ED discharge. The UC San Diego Department of Emergency Medicine faculty provided acute and critical care for nearly 100,000 patient visits annually across two ED locations in San Diego and Imperial Counties. The medical charts are structured through an electronic medical system, data were retrieved using the EPIC electronic query tool. This study was approved by the IRB (Protocol # 803755). This study follows the Strengthening the Reporting of Observational Studies in Epidemiology (STROBE) reporting guidelines for cohort studies (Appendix 1). A trained research associate abstracted the identification of eligible subject data and outcomes electronically using the EPIC electronic query tool. Additionally, two independent abstractors manually abstracted missing data for subjects in the experimental group.

### 2.2. Study participants

**Fig. 1.**
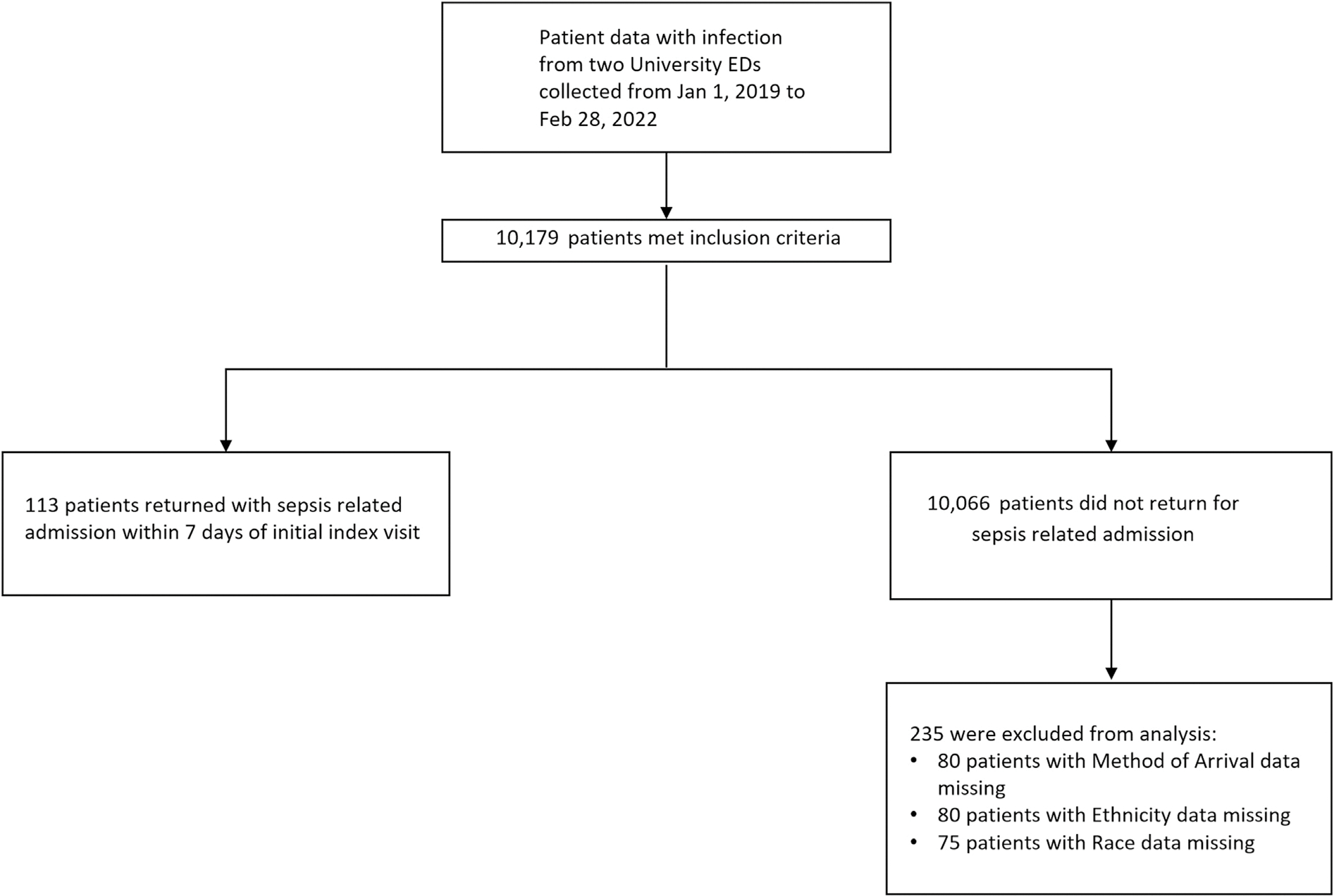
Flow chart of patient selection.

This study focused on infections that were the most common precursors to sepsis. The sepsis diagnosis was determined based on the treating physician’s discretion, reflected by the ICD-10-CM diagnosis. The inclusion criteria encompassed adult patients (aged >/= 18 years) who were discharged from the ED at their index visit with discharge diagnosis (ICD 10-CM code) of pneumonia, urinary tract infection (UTI), and/or cellulitis and who returned for hospital admission within 7 days of the index visit due to sepsis, severe sepsis without septic shock, and/or septic shock (Fig. 1). The index visit was the initial visit during which a patient is seen in the ED and discharged home with an infection diagnosis. We excluded patients who were not discharged home after their initial index visit and patients with missing ethnicity, and race data since all of these patients were in the control group.

### 2.3. Measurements

The primary exposure variables included risk factors for sepsis identified in previous literature and were divided into five categories: (1) demographics: age, sex, race, and ethnicity, (2) comorbidities involving cardiovascular risk factors: hypertension, hyperlipidemia, diabetes, obesity, and cigarette use, (3) chronic medical conditions: peripheral arterial disease, chronic kidney disease, stroke, chronic pulmonary disease, and cancer, (4) proxy of social determinants of health: type of insurance and method of arrival and (5) vital signs: systolic and diastolic pressure, heart rate, respiratory rate, temperature, and oxygen saturation. Obesity was identified in subjects with body mass index (BMI) ≥ 30. For hypertension, we included patients with ICD10 codes that were associated or if they were prescribed an antihypertensive medication. Cardiovascular disease (CVD) included patients with coronary artery disease and myocardial infarction. Diabetes patients were reflected by ICD10 code with diabetes or if they are on a diabetic medication. This was similar to cardiovascular risks and chronic medical conditions that were abstracted based on ICD-10 codes.

### 2.4. Outcome measures

The primary outcome was hospital admission related to sepsis within 7 days of the index visit. We identified patients who developed sepsis based on the admitting diagnosis determined by the treating physician. Sepsis definition encompassed hospital admission diagnosis with ICD 10 codes pertaining to sepsis, severe sepsis, and septic shock.

### 2.5. Statistical analysis and modeling

We compared demographics, comorbidities involving cardiovascular risk factors, chronic medical conditions, proxy of social determinants of health, and vital signs between the cohort of patients who returned for sepsis admission and those who did not return within 7 days of initial infection diagnosis. Histograms were created to assess the normality of the distribution of data. For normally distributed data, *t*-tests were used for continuous variables and the chi-square test for categorical variables. Data were presented as mean +/− standard deviation. The Mann-Whitney *U* Test assessed the difference between the median and interquartile range determined for nonparametric variables.

Demographic characteristics, including age, sex, race, ethnicity, vital signs, a proxy for social determinants of health, comorbidities, cardiovascular risk factors, and chronic medical conditions, were entered into the model. A correlation matrix table was created to assess collinearity. Variance inflation factor, eigenvalue, and eigenvectors of the correlation or covariance were also calculated. No multicollinearity was identified among the independent variables. Since missing data only remained from our control group, we did not include those patients with missing ethnicity and race data.

A sensitive analysis was performed to ensure no selection bias was introduced when excluding cohorts with missing data. There were no significant changes in the association between exposure and outcome variables when excluding cohorts with missing data. Lastly, we used logistical regression to build a model to predict outcome. All analysis were conducted using SAS studio. A *P*-value of <0.05 was considered statistically significant.

Logistical regression was used to predict the probability or odds of subsequent sepsis admission within 7 days of index visit. We used backward stepwise regression excluding variables with *p*-values >0.10 from being included in the final model to prevent over fitting. The predictive and discriminative ability of the model was assessed using the c-statistic, which is defined as the proportion of times the model correctly distinguishes a randomly selected pair of individuals—one with the event and one without. This measure is equivalent to the area under the receiver operating characteristic (ROC) curve. A c-statistic of 0.5 indicates that the model performs no better than random guessing, akin to flipping a coin. A c-statistic of 0.7 to 0.8 suggests good discriminative ability, while a value of 0.8 or higher indicates excellent discriminative ability. Additionally, the Hosmer-Lemeshow test was used to evaluate the model’s goodness of fit.

**Fig. 2.**
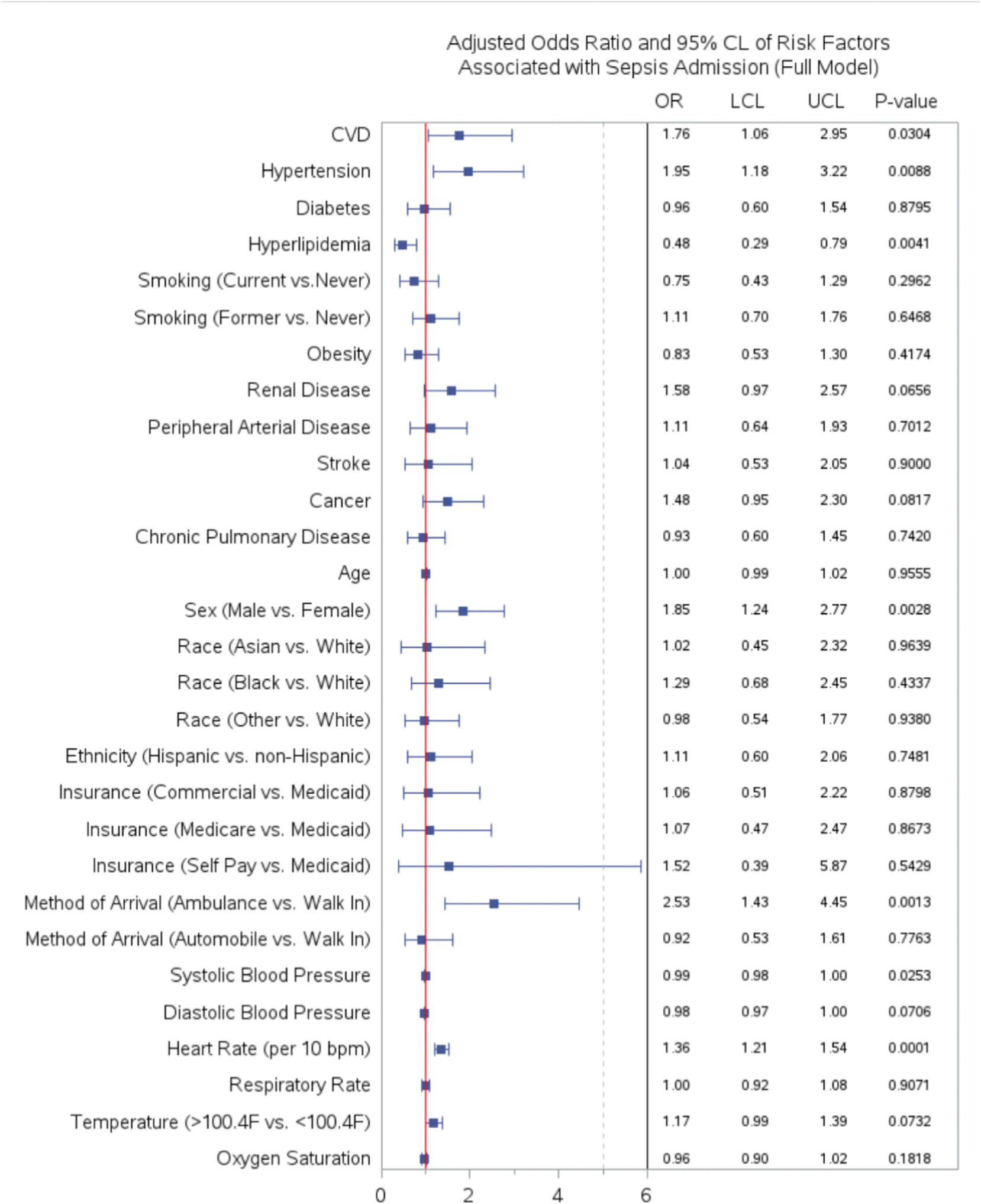
Adjusted Odds Ratio of Risk Factors Associated with Sepsis Admission (Full Model). We examined the association between the selected candidate risk factors and patients with subsequent sepsis admission presenting to the ED with suspected infection. In this full logistic regression model, we evaluated risk factors related to cardiovascular risk factors, chronic medical conditions, type of medical insurance, method of arrival as a proxy for social determinants of health, and vital signs at triage. Points and error bars indicate the odds ratio, 95 % confidence intervals, and *p*-value of variables, respectively.

**Fig. 3.**
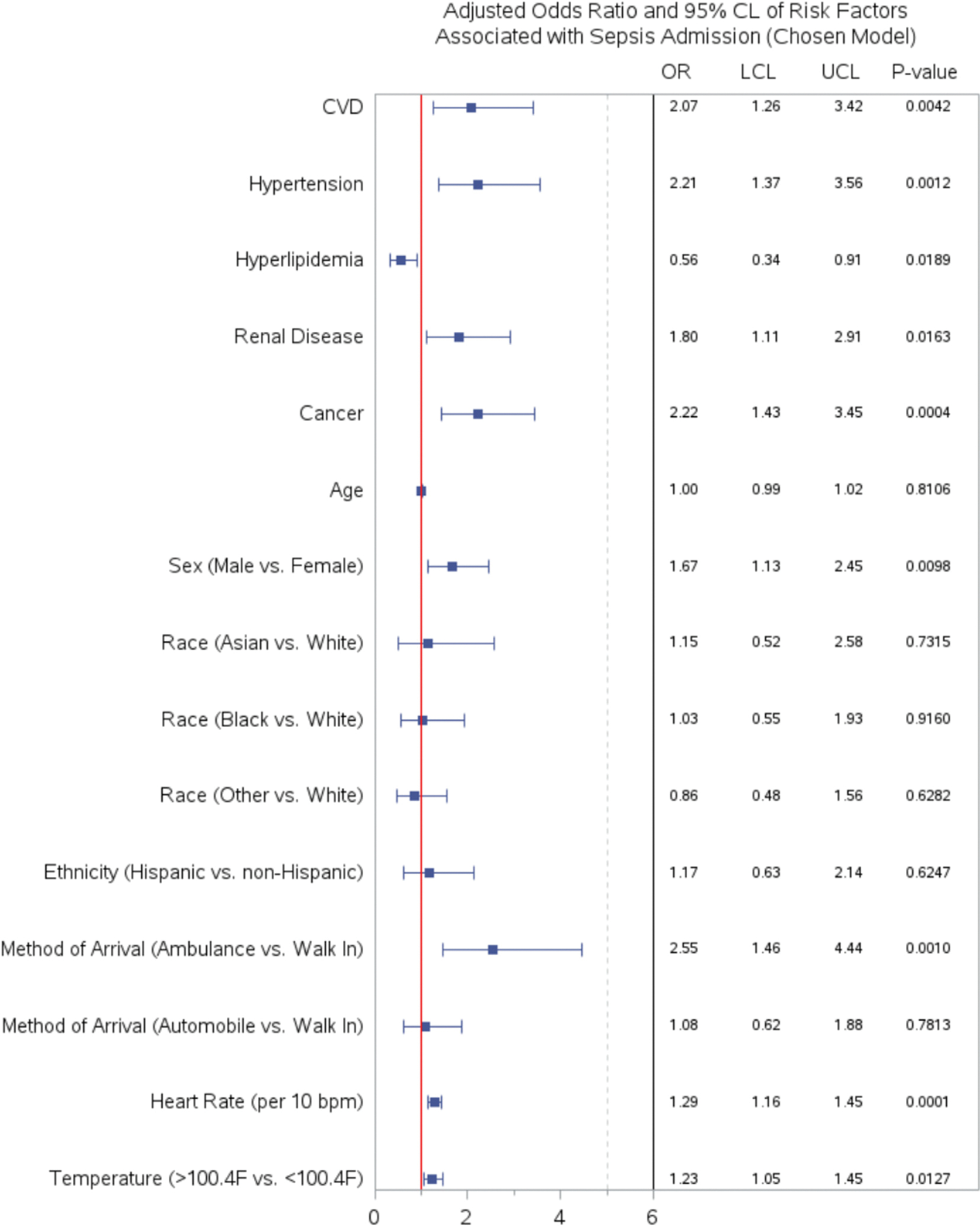
Adjusted Odds Ratio of Risk Factors Associated with Sepsis Admission (Chosen Model). We examined the association between the selected candidate risk factors and patients with subsequent sepsis admission presenting to the ED with suspected infection. We selected the model with relevant risk factors that provided the highest AUC with the best fit. Points and error bars indicate the odds ratio, 95 % confidence intervals, and *p*-value of variables, respectively.

To assess the predictive power of different independent variables, we configured five different models from most basic to most comprehensive. Model 1 is the most basic models containing statistically significant cardiovascular risk factors and chronic medical conditions. Model 2 includes Model 1 and method of arrival. Model 3 includes Model 2 and vital signs. Model 4 is our chosen model. (Fig. 3) Model 5 is the most comprehensive involving all variables considered. (Fig. 2).

## 3. Results

### 3.1. Patient characteristics

Among the 10,179 unique patients at our two study sites, the prevalence of returned sepsis admission within 7 days of the initial index ED visit was 1.11 % (*n* = 113). (Fig. 1) Of the 113 patients who returned for sepsis-related admission, 47 % of patients who returned for sepsis had UTI, 38 % had pneumonia, and 19 % had cellulitis.

### 3.2. Comparison between patients who returned with sepsis admission vs. those who did not return

Patients returned for sepsis admission were likely to be older (mean age of 57 vs. 52 years; Table 1), with a lower ED severity index, and more likely to arrive by ambulance. Of the 113 patients who returned with sepsis admission, 59 % were male. Patients who were admitted with sepsis within 7 days of initial infection diagnosis were more likely to have cardiovascular risk factors and chronic medical conditions. They tended to have CVD (with sepsis 27 % vs. without sepsis 12 %), hypertension (with sepsis 68 % vs. without sepsis 44 %), chronic kidney disease (with sepsis 26 % vs. without sepsis 11 %), and cancer (with sepsis 33 % vs. without sepsis 17 %). There were also differences in vital signs at the initial index visit during triage. Patients who were admitted with sepsis within 7 days have lower systolic blood pressure (mean SBP 129 mmHg vs. 136 mmHg), diastolic pressure (mean DBP 75 mmHg vs. 80 mmHg), higher HR (mean HR 96 bpm vs. 89 bpm), higher temperature (median Temp 98.4F vs. 98.3F), and lower oxygen saturation (median O2 saturation 97 % vs. 98 %).

### 3.3. Risk factors of sepsis among patients with infection at the index visit

The risk factors significantly associated with sepsis among patients with infection were CVD, hypertension, hyperlipidemia, male sex, arriving in an ambulance, and higher heart rate. (Fig. 3). Cardiovascular disease was associated with an odds ratio of 2.07 (95 % CI 1.26–3.42) for return sepsis admission after adjusting for other variables in the final chosen model. Other risk factors associated with sepsis admission include hypertension (OR 2.21 95 % CI 1.37–3.56) and chronic kidney disease (OR 1.80 95 % CI 1.11–2.91). Males had 1.67 times the odds of sepsis related admission(95 % CI 1.13–2.45) compared to females and hyperlipidemia appeared protective (OR 0.56, 95 % CI 0.34–0.91). Additionally, a 10 beats/min increase in heart rate increased the odds of admission for sepsis by 29 %. (OR 1.29 95 % CI 1.16–1.45).

### 3.4. Establishment of a prediction model

**Fig. 4.**
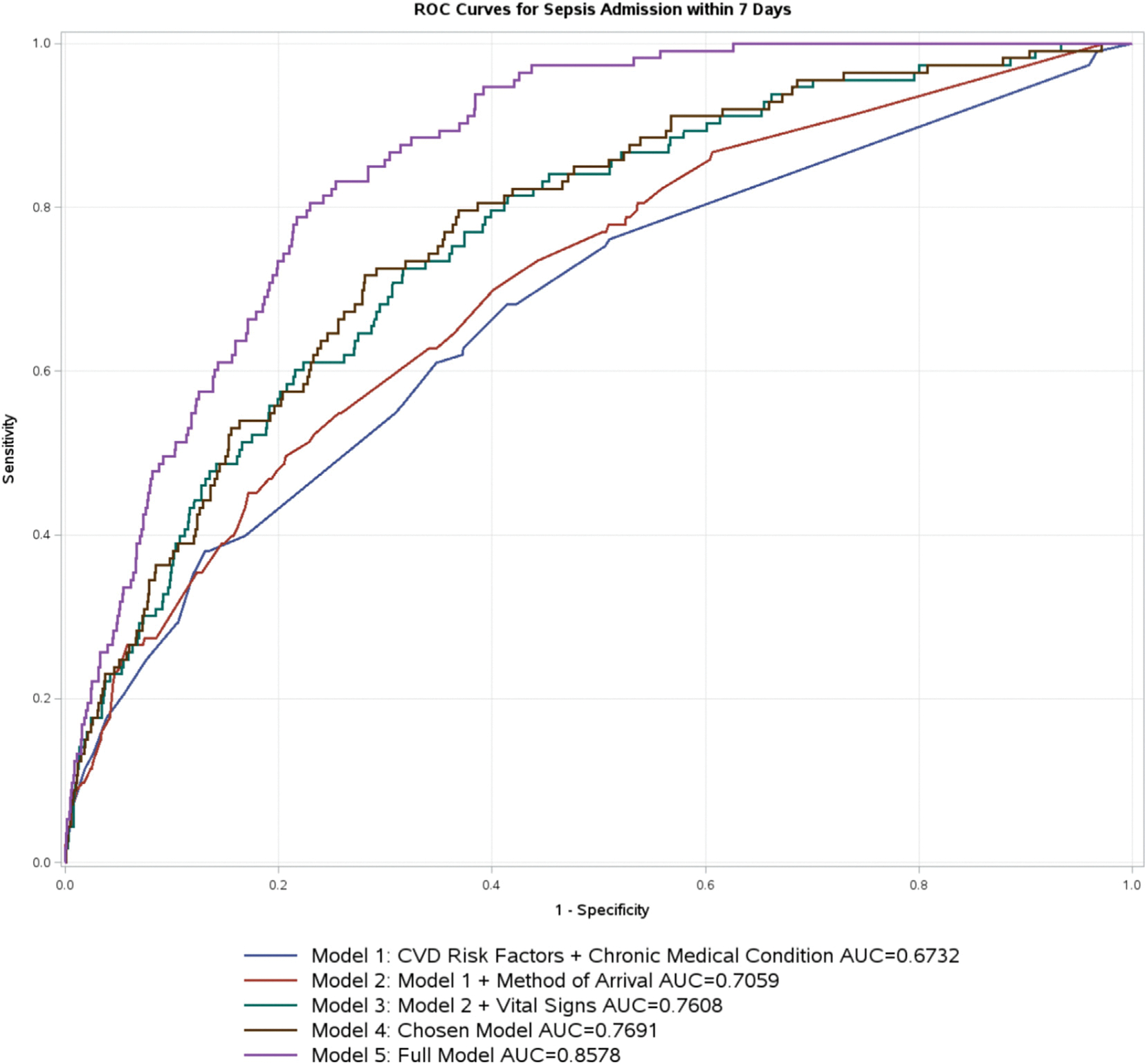
ROC Curves for Sepsis Admission within 7 Days of Initial Infection Diagnosis.

In predicting subsequent sepsis admission, the first model included cardiovascular risk factors and chronic medical conditions. The c-statistic were 0.67 (95 % CI 0.62–0.73). For model 2, we added method of arrival to the first model. The c-statistic improved to 0.71 (95 % CI 0.66–0.75). Heart rate and temperature were added in model 3, which further improved the c-statistic to 0.76 (95 % CI 0.72–0.80). Our chosen model (model 4) has the highest c statistic of the four models with c-statistic of 0.77 (95 % CI 0.73–0.81). Even though model 5 had even higher c-statistic but it had 24 variables. The receiving operating characteristic curve of all five models is reported in Fig. 4. The odds ratios for the full model are reported in Fig. 2.

We examined the association between the selected candidate risk factors and patients with subsequent sepsis admission presenting to the ED with suspected infection. In this full logistic regression model, we evaluated risk factors related to cardiovascular risk factors, chronic medical conditions, type of medical insurance, method of arrival as a proxy for social determinants of health, and vital signs at triage. Points and error bars indicate the odds ratio, 95 % confidence intervals, and *p*-value of variables, respectively.

We limited our chosen model to 12 variables instead of 24 variables in the full model to prevent overfitting. The Hosmer-Lemeshow test for our logistic regression model resulted in a chi-square value of 7.28 with 8 degrees of freedom, giving a *p*-value of 0.51. This suggests that our model fits the data well. (Fig. 3).

## 4. Discussion

In this retrospective cohort study, we tested associations between selected risk factors and subsequent sepsis hospital admission. Focusing on pneumonia, UTI, and cellulitis—the three leading infections causing sepsis— our study is unique in examining patients initially discharged home with an infection and had future sepsis-related admissions within 7 days of index ED visit. We targeted patients who were initially discharged home with infection at initial visits. We developed a statistical model that predicts which patients are at risk of subsequent sepsis admission within 7 days of initial infection diagnosis at ED index visit with good accuracy. The strength of our model is our inclusion of variables from broad categories of risk factors previously identified in literature, including cardiovascular risk factors, chronic medical conditions, demographic characteristics, proxy for social determinants of health, and vital signs. Our findings aim to improve sepsis care and management of patients with infection and sepsis through improved sepsis risk detection, stratification, and assessing modifiable and nonmodifiable risks of sepsis. We found that methods of arrival and vital signs significantly increased predictive power.

Consistent with the findings of Wang et al. from the 2012 Regard Cohort study [7], our analysis revealed elevated odds of sepsis development among males and patients with a history of hypertension, cardiovascular disease (CVD), and chronic kidney disease (Fig. 3). Specifically, individuals with CVD or associated risk factors exhibited higher odds of sepsis development. This observation may be attributed to microvascular dysfunction, a feature shared with sepsis patients [8–12]. The underlying endothelial dysfunction in these individuals likely impairs their ability to mitigate oxidative stress during sepsis, providing a plausible mechanism for these findings [13,14]. A critical aspect of the pathophysiology of chronic arterial hypertension involves the disruption of protective nitric oxide secretion by endothelial due to reactive oxygen species. Compared to normotensive controls, hypertensive patients have been found to possess higher levels of reactive oxygen species and reduced antioxidant defenses [15–17]. When hypertensive patients develop an infection, the likelihood of sepsis may increase due to the underlying endothelial dysfunction, characterized by elevated oxidative stress and an increased burden of reactive oxygen species. Similarly, patients with chronic kidney disease may have higher odds of developing sepsis because CKD appeared to be associated with loss of coherent network of microvessels causing a heterogeneous pattern of microvascular rarefaction, diminished microvascular blood flow velocity, and impaired oxygen uptake in tissue [18].

Males in our study had higher odds of future sepsis admissions than females, consistent with previous research showing sex disparities in sepsis. Offner et al. [19] identified male sex as a risk factor for severe infections post-surgery, and another study [20] noted higher pneumonia incidence in male trauma victims. The influence of androgen on immune cells might lead to immunosuppression in males [21,22], while female hormones could offer protective effects [21–23]. A study on community-acquired pneumonia showed older men had lower survival rates with increased pro-inflammatory markers, while females had higher anti-inflammatory responses [24]. Sex hormones, not just sex, are key to these observations; animal studies with flutamide suggest testosterone suppresses inflammation, whereas estradiol enhances immune responses [25]. However, the debate continues on sex-specific impacts on lymphocyte activation and sepsis mortality.

We observed unexpected findings where hyperlipidemia appeared to offer a protective effect against sepsis admissions (Fig. 3), corroborated by meta-analyses from Taylor et al. [26] and Hofmaenner et al. [27], which noted that lower levels of HDL-C, LDL-C, and total cholesterol in sepsis patients correlate with higher mortality. Guiguis et al. [28] also demonstrated that lower HDL-C, ApoA-I demonstrated worsened sepsis outcomes. This inverse relationship between plasma cholesterol levels and inflammation markers has been observed across various patient groups including those critically ill [29,30], post-surgical [30], with major trauma, burns [31,32], viral infections like Covid-19 [33], and cancer patients receiving recombinant TNF-*α* [34]. Several biological mechanisms might explain hypocholesterolemia in sepsis, such as decreased intake, impaired intestinal cholesterol absorption [35] reduced synthesis [36], impaired transport [37,38], and increased metabolism and elimination through toxic scavenging [39,40]. Low cholesterol can negatively affect both innate and adaptive immune responses, as cholesterol is vital for TLR signaling in macrophages [41]. Additionally, cholesterol and lipoproteins can neutralize toxins like endotoxin, preventing TLR activation [42], while oxysterols might influence cytokine production, virus entry, and immune response modulation [43], potentially increasing anti-inflammatory cytokines like IL-10 [44–46]. However, the exact mechanisms linking hypocholesterolemia to sepsis outcomes are still not fully understood, necessitating further research.

Prior evidence suggested that social determinants of health improved the high risk of unplanned 30-day readmission models [47]. Social determinants of health variable such as socioeconomic status, gender, old age, and frailty were found to be associated with sepsis [47,48]. In our study, we used medical insurance and methods of arrival to the ED as proxy for social determinants of health. Patients without medical health insurance and who arrived by ambulance were associated with subsequent sepsis admission. We also considered vital signs as risk factors of sepsis. Similar to Shibata et al. [49], we found higher heart rate, and higher temperature associated with subsequent sepsis development. These vital signs at triage during index visits could be helpful for physicians in identifying patients at high risk for subsequent sepsis admission.

## 5. Limitations

We recognized several potential limitations in our study. First, the research was conducted at two medical centers within the same health system, which may limit the generalizability of our findings. Second, the small sample size of 113 patients with sepsis admission may render many risk factors statistically insignificant, suggesting that our results would benefit from validation in a multicenter study.

Thirdly, patients were given antibiotic prescriptions that required independent filling. Adherence to their antibiotic regimen after the initial ED visit could be a confounding and unmeasurable factor. Additionally, this study relies on retrospective evaluation of electronic health record data, which can suffer from missing information, potentially reducing statistical power, introducing bias in parameter estimation, or diminishing sample representativeness [50]. However, a sensitivity analysis was performed to ensure minimal bias introduced if present. Another challenge of electronic health records is the risk of misclassification due to data entry practices that may not align with the intended use. Specifically, our study used administrative data with ICD-10 codes to define sepsis, CVD, CVD risk factors, and comorbidities, which might lead to misclassification errors. Lastly, there is the possibility that patients could have sought care at a different hospital outside of our healthcare system following their initial visit, potentially underestimating the actual prevalence of return sepsis admissions.

## 6. Conclusion

In this study, we identified risk factors that can be used to predict sepsis related admission for patients who were initially discharged from the ED with pneumonia, UTI, and/or cellulitis. About 1.1 % of the patients returned with sepsis-related admission within 7 days of ED index visit. Risk factors that are associated with subsequent return sepsis admission in our chosen model include CVD, hypertension, chronic kidney disease, cancer, male, arrival by ambulance, and increased heart rate and temperature. Hyperlipidemia was found to be protective. Our findings may be used to risk-stratify and guide outpatient disposition decisions for ED patients with infection and determine which patients need to be more closely monitored in the outpatient setting following ED discharge.

## Supplementary Material

1-s2.0-S0735675725005200-mmc2

1-s2.0-S0735675725005200-mmc1

## Figures and Tables

**Table 1:** Characteristics of patients who did and did not return for sepsis related admission within 7 days of infection.

	Infection with return	Infection without return	*P* Value
			
	sepsis admission	sepsis admission	
			
	(n = 113)	(n = 9831)	

***Age (year), mean (SD)***	57.20 (19.54 %)	52.03 (19.50 %)	0.0050
***Sex, n (%)***			0.0003
*Female*	46 (40.71 %)	5591 (56.87 %)	
*Male*	67 (59.29 %)	4240 (43.13 %)	
***BMI Median (IQR)***	25.1 (22.0–30.3)	26.5 (22.9–31.3)	0.138
***Race***			0.6872
*White*	62 (54.87 %)	5233 (53.23 %)	
*Black*	13 (11.50 %)	926 (9.42 %)	
*Asian*	7 (6.19 %)	583 (5.93 %)	
*Other*	31 (27.43 %)	3089 (31.42 %)	
***Ethnicity***			0.5755
*Hispanic*	27 (23.89 %)	2578 (26.22 %)	
*Non Hispanic*	86 (76.11 %)	7253 (73.78 %)	
***Type of Insurance***			0.0486
*Medicare*	36 (31.86 %)	2234 (21.72 %)	
*Medicaid*	9 (7.96 %)	988 (10.05 %)	
*Commercial*	65 (57.52 %)	5776 (58.75 %)	
*Self Pay*	3 (2.65 %)	562 (5.72 %)	
*Others*	0 (0.00 %)	271 (2.76 %)	
***Method of Arrival***			<0.0001
*Ambulance*	45 (39.82 %)	2133 (21.70 %)	
*Walk In*	16 (14.16 %)	2384 (24.25 %)	
*Private Automobile*	52 (46.02 %)	5314 (54.05 %)	
***ED Severity Index (ESI)***			
*(1 = High Acuity 5 = Low Acuity), mean (SD)*	2.93(0.56)	3.05 (0.51)	0.0138
***Comorbidities***			
*Cardiovascular Vascular Disease (CVD)*	31 (27.43 %)	1141 (11.61 %)	<0.0001
*Hypertension*	77 (68.14 %)	4359 (44.34 %)	<0.0001
*Diabetes*	31 (27.43 %)	2051 (20.86 %)	0.0878
*Hyperlipidemia*	38 (33.63 %)	2757 (28.04 %)	0.1892
*Current Smoker*	24 (21.24 %)	1461 (14.86 %)	<0.0001
*Former Smoker*	37 (32.74 %)	1701 (17.30 %)	
*Never Smoker*	52 (46.02 %)	3650 (37.13 %)	
*Smoker(Unknown Status)*	0 (0.00 %)	3019 (30.71 %)	
*Obesity*	29 (25.66 %)	2797 (28.45 %)	0.5137
***Chronic Medical Condition***			
*Chronic Kidney Disease*	29 (25.66 %)	1072 (10.90 %)	<0.0001
*Peripheral Artery Disease*	19 (16.81 %)	849 (8.64 %)	0.0022
*Stroke*	11 (9.73 %)	577 (5.87 %)	0.0833
*Chronic Pulmonary Disease*	32 (28.32 %)	2161 (21.98 %)	0.1062
*Cancer*	37 (32.74 %)	1645 (16.73 %)	<0.0001
***Vitals Signs, mean (SD)***			
*Systolic Blood Pressure*	128.71 (22.23)	136.44 (22.01)	0.0002
*Diastolic Blood Pressure*	75.09 (13.74)	80.32 (14.52)	0.0001
*Heart Rate*	95.65 (17.03)	88.91 (16.87)	<0.0001
*Respiratory Rate*	17.99 (2.31)	17.59 (2.21)	<0.0001
*Temperature (F), median (IQR)*	98.40 (98.0–99.1)	98.30 (98.0–98.8)	0.0124
*Oxygen Saturation, median (IQR)*	97.0 (96.0–99.0)	98.0 (97.0–99.0)	<0.0001
***Final Diagnosis/Outcome, n (%)***			
*Pneumonia*	43(38.05 %)	1857 (18.89 %)	<0.0001
*UTI*	53(46.90 %)	4579 (46.58 %)	0.9540
*Cellulitis*	21(18.58 %)	3481 (35.41 %)	0.0004

## Data Availability

The entire deidentified dataset, data dictionary and analytic code for this investigation are available upon request, from the date of article publication by contacting Alice Y. Chen MD,MPH at alc022@health.ucsd.edu.

## Appendix A. Supplementary data

Supplementary data to this article can be found online at https://doi.org/10.1016/j.ajem.2025.07.059.
