# Supplementary material for: Risk factors associated with return sepsis admission following emergency department discharge with infection
Source: Am J Emerg Med. Author manuscript; Available in PMC 2026 Feb 24. (PMC12930701; doi:10.1016/j.ajem.2025.07.059)
Supplement: 1-s2.0-S0735675725005200-mmc2 [file NIHMS2135633-supplement-1-s2_0-S0735675725005200-mmc2.docx]

**Appendix 2: ICD 10 Codes**

| CVD | I25x |
| --- | --- |
| Hypertension | I10x, I15x |
| Hyperlipidemia | E78x |
| Diabetes | E08x-E13x |
| Cigarette Use | Obtained from Social History |
| Peripheral Arterial Disease | \| I70x,I71x,I73.1x,I73.8x, I73.9x, \| \| --- \| \| I77.1x,I79.0x,I79.2x,K55.1x, \| \| K55.8x,K55.9x,Z95.8x,Z95.9x \| |
| Chronic Kidney Disease | \| I12.0x,I13.0x,N03.2x-N03.7x, \| \| --- \| \| N05.2x-N05.7x,N18x,N19x, \| \| N25.0x,Z49.0x-.1x,Z94.2x,Z99.2x \| |
| Chronic Pulmonary Disease | \| I27.8x,I27.9x,J41x-J47x, \| \| --- \| \| 60x-J67x,J68.4,J70.1x,  J70.3x \| |
| Cancer | \| Cx,  D46x,D47x \| \| --- \| \| C81x-C86x \| \| C91x-C95x \| |
| Obesity | Calculated from BMI |
| Stroke | I63x,I65x,I67x |
| Pneumonia | B57, J10, J13, J14, J15, J16, J18,.9, J69 |
| UTI | N10, N30.00, N30.01, N34.2, N39.0 |
| Cellulitis | L03.011,L03.012,L03.031,L03.032,L03.039,L03.111, L03.112, L03.113, L03.114, L03.115, L03.116, L03.119, L03.211, L03.213, L03.221, L03.311,L03.312,L03.314, L03.315,L03.316, L03.317, L03.319,L03.811,L03.818,L03.90) |
| Sepsis | A02.1,A22.7,A24.1 A26.7,A32.7,A40 A41,A42.7,A54.86, B37.7,O03.37, O03.87 O04.87, O07.37, O08.82, O85, O86.04, P36.8, T81.44, T88.0 |
| Severe Sepsis | R65.20 |
| Septic Shock | R65.21 |
